# Hepatitis C Virus Testing in Perinatally Exposed Children

**DOI:** 10.1001/jamanetworkopen.2026.0743

**Published:** 2026-04-17

**Authors:** Rachel L. Epstein, Sarah Munroe, Elizabeth A. Erdman, Amy Bettano, Megan R. Curtis, Laura White, Elisha M. Wachman, Anthony Osinski, Alexander Y. Walley, Benjamin P. Linas

**Affiliations:** 1 Division of Infectious Diseases, Department of Medicine, Boston University Chobanian and Avedisian School of Medicine, Boston, Massachusetts; 2 Section of Infectious Diseases, Boston Medical Center, Boston, Massachusetts; 3 Office of Population Health, Massachusetts Department of Public Health, Boston; 4 Division of Infectious Diseases, Washington University School of Medicine, St Louis, Missouri; 5 Department of Biostatistics, Boston University School of Public Health, Boston, Massachusetts; 6 Section of Newborn Medicine, Department of Pediatrics, Boston University Chobanian and Avedisian School of Medicine, Boston, Massachusetts; 7 Bureau of Infectious Disease and Laboratory Sciences, Massachusetts Department of Public Health, Boston,; 8 Clinical Addiction Research and Education Unit, Section of General Internal Medicine, Department of Medicine, Boston University Chobanian and Avedisian School of Medicine, Boston, Massachusetts; 9 Bureau of Substance Addiction Services, Massachusetts Department of Public Health, Boston

## Abstract

**Importance:**

Despite rising hepatitis C virus (HCV) incidence in pregnant people, studies demonstrate that fewer than 50% of perinatally exposed infants receive HCV testing. Few studies report treatment of infected infants or factors affecting testing.

**Objective:**

To identify birthing parent and child factors associated with appropriate HCV testing for perinatally exposed infants and characterize HCV care for children with HCV.

**Design, Setting, and Participants:**

This cohort study included records on testing, care, and treatment for Massachusetts children perinatally exposed to HCV between 2014 and 2021 from a statewide health database linking statewide vital statistics, infectious disease surveillance data, substance use treatment, and insurance claims data. Data were analyzed from April 2023 to June 2025.

**Exposure:**

Linkage to a birthing parent with a confirmed (RNA positive) or probable (antibody positive; no RNA testing) HCV case reported to the Massachusetts Department of Public Health.

**Main Outcomes and Measures:**

Guideline-appropriate HCV antibody or RNA testing and HCV treatment were the main outcomes. Multivariable logistic regressions were used to analyze factors associated with guideline-concordant HCV testing.

**Results:**

Between 2014 and 2021, 4548 children were born (between 0.7% and 1.3% of yearly births) in Massachusetts to 3693 birthing parents with probable or confirmed HCV; among 4103 children who were aged 2 years or older by study end (2103 male [51.3%]; 289 Black non-Hispanic [7.0%], 575 Hispanic [14.0%], 3069 White non-Hispanic [74.8%]; 3185 [77.6%] with public insurance at delivery), 41.9% completed appropriate perinatal HCV exposure testing. HCV was diagnosed in 40 (2.5%) of those tested; between 1 and 10 of 38 treatment-eligible children were treated by 2022 (number suppressed due to value between 1 and 10). Appropriate testing rates decreased over time and were lowest for those born in 2020 (32.3%; 95% CI, 28.9%-35.7%). A medical claim for the birthing parent’s HCV within 30 days of delivery (adjusted odds ratio [aOR], 5.40; 95% CI, 4.19-6.96), medication for opioid use disorder treatment during pregnancy (aOR, 1.29; 95% CI, 1.09-1.51), and factors correlated with HCV risk were associated with significantly higher odds of appropriate testing; non-Hispanic Black race (aOR, 0.65; 95% CI, 0.45-0.94) and private insurance (aOR, 0.76; 95% CI, 0.62-0.93) were associated with lower appropriate testing odds.

**Conclusions and Relevance:**

In this cohort study of perinatally exposed infants, HCV testing decreased over time, and few were treated. Communication and recognition of the birthing parent’s HCV risk or diagnosis were associated with greater odds of testing completion; these modifiable factors could be addressed to improve equitable HCV testing and care.

## Key Points

**Question:**

What factors are associated with hepatitis C virus (HCV) testing for infants perinatally exposed, and what proportion of infants with perinatal HCV infection are linked to care and treated?

**Findings:**

In this cohort study including 4548 children born to 3693 birthing parents with probable or confirmed HCV, only 42% of perinatally-exposed infants were tested; testing declined over the course of the study period. Infants of birthing parents with public insurance, known HCV risk factors, or an HCV diagnosis claim during delivery hospitalization had higher testing odds.

**Meaning:**

These results suggest that communication between perinatal and pediatric clinicians and routine assessment for HCV exposure in all children will be critical to successfully implement perinatal HCV testing guidance and improve pediatric HCV care.

## Teaser

This cohort study of US infants perinatally exposed to hepatitis C virus estimates the proportion who receive guideline-appropriate testing and treatment and examines characteristics associated with receipt of testing.

## Introduction

Hepatitis C virus (HCV) prevalence has risen 10-fold among pregnant persons since 2000, to 4.9 cases per 1000 live deliveries in 2019.^1^ Perinatal infection occurs in approximately 8% of exposed infants, and is the most common HCV transmission mode among children.^2^

In 1998, the Centers for Disease Control and Prevention (CDC) and American Academy of Pediatrics recommended HCV antibody testing of all children born to birthing parents with active HCV infection at age 18 months; maternal HCV antibody can persist until then, and RNA testing is more expensive.^3,4,5,6^ Diagnosis before 18 months would not result in earlier treatment; effective HCV cure (direct-acting antivirals [DAAs]) were approved for 12-to-17-year-olds in 2017, for 3-to-11-year-olds in 2019, and pangenotypic DAAs for children aged 3 years or older in June 2021^7,8,9^; no HCV treatment has been FDA-approved for children under 3 years old. Under these guidelines, only an estimated 30.1% (range, 8.6%-53.1%) of exposed infants were appropriately tested for HCV, largely due to loss to follow-up.^3^

In October 2023, the CDC recommended RNA testing for infants exposed to HCV between ages 2 and 6 months to reduce loss to follow-up before later antibody testing.^3^ While we await data on testing practices following the new guidelines, understanding factors associated with improved testing could shape interventions to implement these recommendations.

Previous studies demonstrated improved infant testing when birthing parents accessed health care for their own HCV, HIV, or substance use care and with outpatient infant HCV exposure documentation.^10,11,12,13,14^ Studies indicate contradictory results about testing disparities by race, ethnicity, insurance, or foster care. For example, while some studies observed decreased testing in Black or Hispanic children compared with non-Hispanic White children,^14,15,16^ others showed increased testing in Black and/or Hispanic children^11,17^ or no difference by race or ethnicity.^11,18^ These studies lacked the sample size or sociodemographic covariates to robustly adjust for confounders.

We used a statewide population-level data warehouse that allows linkage of birthing parent-infant dyads, medical and pharmacy claims, infectious disease reporting data, and sociodemographic information to characterize perinatal HCV exposure, testing, and treatment over time in Massachusetts. These data can identify populations with lower perinatal HCV testing and quantify treatment gaps to better tailor interventions to reach national 2030 HCV elimination goals.^19^

## Methods

### Overview

We used the Massachusetts Public Health Data Warehouse (PHD), a statewide database maintained by the Massachusetts Department of Public Health (DPH), to characterize trends in perinatal HCV exposure, testing, and care in Massachusetts. We analyzed factors associated with HCV testing in infants perinatally exposed to HCV. This work was mandated by law and conducted by a public health authority. The Massachusetts Department of Public Health was not engaged in human participants research, and no institutional review board approval was required. This study followed the Strengthening the Reporting of Observational Studies in Epidemiology (STROBE) reporting guideline for cohort studies (eTable 4 in Supplement 1).

### Dataset

The PHD contained 30 administrative data sources that could be linked at the individual-level, including the Massachusetts All-Payer Claims Database (APCD), vital statistics data (birth and death records), HCV case reports, prescription records from the Prescription Monitoring Program, and substance use treatment data from the Bureau of Substance Addiction Services (BSAS) (eMethods in Supplement 1).^20,21^ All data are deidentified, only tabular or summary data are visible to investigators, and values from 1 through 10 and complementary values are suppressed per DPH privacy requirements to prevent calculation of suppressed values. This study was approved by the Boston University Medical Campus institutional review board as not human participants research.

### Cohort

We identified birthing parents who had a probable (positive HCV antibody without any RNA testing) or confirmed (positive RNA) HCV case reported to the DPH between 2011 and 2021.^22^ We linked birthing parents to all subsequent liveborn infant deliveries from 2014 through 2021. We excluded children with a birthing parent prescribed HCV treatment prior to delivery as those infants may not have been exposed to HCV, an insurance plan that did not report to the APCD after 2016, no APCD claims, or who died before 18 months. For testing analyses, we included children born from 2014 to 2020 who would be 2 years old and eligible for guideline-based HCV testing (antibody testing after 18 months) by December 2022 (study end). For linkage to care and treatment, we included children born by June 2019 and eligible for treatment (age 3 years or older) for at least 6 months before December 2022.

### Outcomes

To determine HCV prevalence among pregnant people and testing, linkage, and treatment of HCV-exposed children over time, we searched the APCD medical and pharmacy datasets for HCV-related testing, diagnoses, and treatments (eTables 1-2 in Supplement 1). We defined the following stages of the pediatric HCV care cascade: (1) any HCV testing (presence of any HCV antibody or RNA test at any age), (2) appropriate HCV testing (primary outcome; an HCV RNA test completed at age 2 months or older or an HCV antibody test done at 18 months or later), and (3) HCV infection (any child with a confirmed HCV case reported to the DPH at younger than age 3 years).^22,23^ For children infected with HCV, we measured linkage to care (a visit claim with a primary encounter diagnosis of HCV using *International Classification of Diseases, Ninth Revision (ICD-9)* and *International Statistical Classification of Diseases and Related Health Problems, Tenth Revision (ICD-10)* codes) and treatment (any DAA claim) (eTable 2 in Supplement 1).

### Statistical Analysis

We used descriptive statistics to summarize care cascade outcomes for children exposed to HCV. HCV case report data were available for 2011 to 2021; all other included datasets spanned 2014 to 2022. Therefore, data pertaining to children with HCV include cases identified through 2021; testing, care, and treatment reflect medical and pharmacy claims through 2022. We calculated 95% CIs for yearly prevalence of infants exposed to HCV and proportion appropriately tested using the standard formula for proportions and antibody and RNA testing over time using a Cochran-Armitage trend test. We compared age at first HCV test over time using a nonparametric Kruskal-Wallis test.

We used logistic regression to analyze factors associated with appropriate HCV testing in children exposed to HCV, adjusting for baseline covariates (eTable 2 in Supplement 1). Our primary exposures of interest were those with inconsistent associations with appropriate testing in previous research: birthing parent’s race, Hispanic ethnicity, and insurance. As race is not a biological construct but rather a marker for socioeconomic risk and experiences of structural racism, we hypothesized that adjusting for additional sociodemographic factors (including education, homelessness, incarceration, residential county), health care engagement factors (prenatal care adequacy, well-care visits, medication for opioid use disorder [MOUD] treatment), and birthing parent comorbidities (eg, substance use, HIV) could uncover previously unmeasured confounders of this association. We used race and Hispanic ethnicity categories from the spine demographics table, which uses a tiered algorithm to search 25 PHD datasets to determine the most accurate categories, prioritizing those with self-reported values. The other category varies by dataset and includes any non-Hispanic race that is not White, Black, or Asian. We used generalized estimation equations (GEE) to fit the model with clustered standard errors by birthing parent to account for multiple births to the same parent.

**Figure 1.**
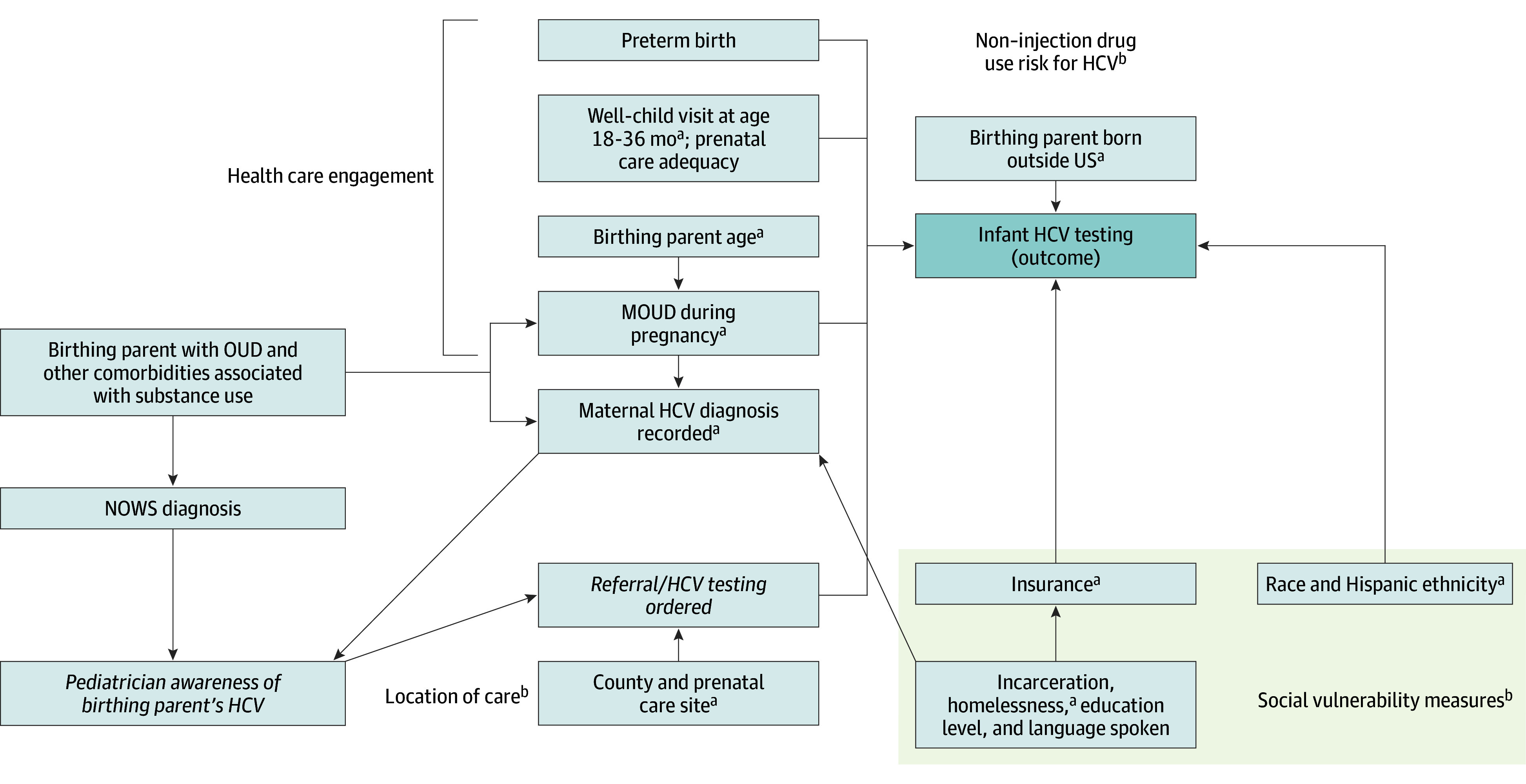
Directed Acyclic Graph for Regressions HCV indicates hepatitis C virus; MOUD, medication for opioid use disorder; NOWS, neonatal opioid withdrawal syndrome. Italics represent variables not capturable in the dataset that represent key steps other variables may lead to. ^a^Variables ultimately selected for multivariable analysis based on statistically significant associations in the univariable analysis, after checking for collinearity, removing those too highly correlated with other variables, and checking fit statistics, with and without inclusion of specific variables. ^b^Words outside boxes denote categories of variables and why they were included. Social vulnerability measures include insurance; race and Hispanic ethnicity; and incarceration, homelessness, education level, and language spoken.

Multivariable models included covariates suspected a priori to be associated with the outcome (appropriate HCV testing) and significant in the univariable analysis. To test for collinearity, we calculated χ^2^ tests between variables, categorized similar variables, and created a directed acyclic graph to describe associations (Figure 1). We chose 1 to 2 representative variables for each group of similar covariates, balancing clinical relevance, significance in unadjusted analysis, and model fit statistics.

To mitigate effects of missing children who may have moved out of state before completing testing, we performed sensitivity analyses using complete case regressions for infants who had a captured well-care visit between 18 and 36 months of age. We analyzed data using a 2-sided *P* < .05 threshold for significance using SAS Studio version 3.81 (SAS Institute Inc) between April 2023 and June 2025.

## Results

From 2014 to 2020, 4103 infants were born to 3419 unique birthing parents with HCV (2103 male [51.3%]; 289 non-Hispanic Black [7.0%], 575 Hispanic [14.0%], and 3069 non-Hispanic White [74.8%]) (Table 1). In the full cohort, 662 births (16.1%) were preterm, 599 infants (14.6%) had a diagnosis of neonatal opioid withdrawal syndrome (NOWS), and 3437 infants (83.8%) had 1 or more well-child visits between ages 18 to 36 months.

**Table 1.** Infants Born to Birthing Parents With Probable or Confirmed Hepatitis C Virus (HCV), 2014-2020

Characteristics	Infants, No. (%)		
	Total (N = 4103)	Appropriately tested^a^ (n = 1718)	Not appropriately tested^a^ (n = 2385)
**Infants**			
Sex			
Female	2000 (48.7)	844 (49.1)	1156 (48.5)
Male	2103 (51.3)	874 (50.9)	1229 (51.5)
Race and ethnicity			
American Indian or other, non-Hispanic^b^	90 (2.2)	31 (1.8)	59 (2.5)
Asian or Pacific Islander, non-Hispanic	65 (1.6)	13 (0.8)	52 (2.2)
Black, non-Hispanic	289 (7.0)	111 (6.5)	178 (7.5)
Hispanic	575 (14.0)	213 (12.4)	362 (15.2)
White, non-Hispanic	3069 (74.8)	1347 (78.4)	1722 (72.2)
Missing	15 (0.4)	NR^c^	NR^d^
Residential county at birth			
Barnstable	282 (6.9)	117 (6.8)	165 (6.9)
Berkshire	117 (2.9)	38 (2.2)	79 (3.3)
Bristol	606 (14.8)	270 (15.7)	336 (14.1)
Dukes	NR^c^	NR^c^	NR^c^
Essex	460 (11.2)	172 (10.0)	288 (12.1)
Franklin	62 (1.5)	22 (1.3)	40 (1.7)
Hampden	263 (6.4)	99 (5.8)	164 (6.9)
Hampshire	66 (1.6)	30 (1.7)	36 (1.5)
Middlesex	633 (15.4)	272 (15.8)	361 (15.1)
Nantucket	NR^c^	NR^c^	NR^c^
Norfolk	303 (7.4)	147 (8.6)	156 (6.5)
Plymouth	448 (10.9)	197 (11.5)	251 (10.5)
Suffolk	325 (7.9)	153 (8.9)	172 (7.2)
Worcester	526 (12.8)	198 (11.5)	328 (13.8)
Preterm birth			
Yes	662 (16.1)	290 (16.9)	372 (15.6)
No	3434 (83.7)	1427 (83.1)	2007 (84.2)
Unknown	NR^c^	NR^c^	NR^c^
Diagnosis of NOWS in birth data			
Yes	599 (14.6)	279 (16.2)	320 (13.4)
No	3082 (75.1)	1279 (74.4)	1803 (75.6)
Unknown	422 (10.3)	160 (9.3)	262 (11.0)
Any WCC captured between 18-36 mos of age			
Yes	3437 (83.8)	1649 (96.0)	1788 (75.0)
No	666 (16.2)	69 (4.0)	597 (25.0)
**Birthing parent**			
Age at delivery, mean (SD), y	29.1 (4.9)	29.4 (5.04)	28.7 (4.65)
Age group at delivery			
≤18 y	17 (0.4)	NR^c^	NR^d^
19-25 y	991 (24.2)	443 (25.8)	548 (23.0)
26-35 y	2654 (64.7)	1126 (65.5)	1528 (64.1)
>35 y	440 (10.7)	144 (8.4)	296 (12.4)
Unknown	NR^c^	0	NR^c^
Race and ethnicity			
Asian or Pacific Islander, American Indian, or other, non-Hispanic^b^	90 (2.2)	20 (1.1)	70 (2.9)
Black, non-Hispanic	226 (5.5)	72 (4.2)	154 (6.5)
Hispanic	324 (7.9)	107 (6.2)	217 (9.1)
White, non-Hispanic	3463 (84.4)	1520 (88.5)	1943 (81.5)
Born outside the US			
Yes	288 (7)	69 (4.0)	219 (9.2)
No	3813 (92.9)	1649 (96.0)	2164 (90.7)
Unknown	NR^d^	NR^c^	NR^d^
Insurance at delivery			
Private	874 (21.3)	247 (14.4)	627 (26.3)
Public	3185 (77.6)	1450 (84.4)	1735 (72.7)
Unknown	44 (1.1)	21 (1.2)	23 (1.0)
Language spoken			
English	3920 (95.5)	1659 (96.6)	2261 (94.8)
Spanish or other	112 (2.7)	28 (1.6)	84 (3.6)
Refused or unknown	71 (1.7)	31 (1.8)	40 (1.7)
Highest level of education			
No high school degree	707 (17.2)	306 (17.8)	401 (16.8)
High school degree or GED	1402 (34.2)	640 (37.3)	762 (31.9)
Associate or Bachelor’s degree	443 (10.8)	133 (7.7)	310 (13.0)
Post graduate education	129 (3.1)	13 (0.8)	116 (4.9)
Other or unknown	1422 (34.7)	626 (36.4)	796 (33.4)
Ever experienced homelessness or housing instability^e^			
Yes	3186 (77.6)	1503 (87.5)	1683 (70.6)
No	759 (18.5)	207 (12.0)	552 (23.1)
Unknown or missing	158 (3.9)	NR^c^	NR^d^
Ever incarcerated			
Yes	1530 (37.3)	726 (42.3)	804 (33.7)
No	2573 (62.7)	992 (57.7)	1581 (66.3)
**Clinical or birth data**			
Kotelchuck Index^f^			
Inadequate (<50% of expected visits)	1105 (26.9)	532 (31.0)	573 (24.0)
Intermediate (50%-79%)	447 (10.9)	174 (10.1)	273 (11.4)
Adequate (80%-109%)	1141 (27.8)	430 (25.0)	711 (29.8)
Intensive (≥110%)	1218 (29.7)	488 (28.4)	730 (30.6)
Missing or unknown	192 (4.7)	94 (5.5)	98 (4.1)
Prenatal care site			
Community health center	306 (7.5)	138 (8.0)	168 (7.0)
HMO	103 (2.5)	39 (2.3)	64 (2.7)
Private physician’s office	2583 (63.0)	1034 (60.2)	1549 (64.9)
Hospital clinic	886 (21.6)	412 (24.0)	474 (19.9)
Other	41 (1.0)	20 (1.2)	21 (0.9)
Unknown	184 (4.5)	75 (4.4)	109 (4.6)
Diagnosis of HBV in birth data			
Yes	47 (1.1)	17 (1.0)	30 (1.3)
No	4055 (98.8)	1700 (99.0)	2355 (98.7)
Unknown	NR^c^	NR^c^	NR^c^
Diagnosis of HCV in birth data			
Yes	2304 (56.2)	1166 (67.9)	1138 (47.7)
No	1798 (43.8)	551 (32.1)	1247 (52.3)
Unknown	NR^c^	NR^c^	NR^c^
Diagnosis of HCV recorded during delivery hospitalization			
Yes	3264 (79.6)	1617 (94.1)	1673 (70.1)
No	839 (20.4)	101 (5.9)	712 (29.9)
Diagnosis of HIV			
Yes	16 (0.4)	NR^c^	NR^d^
No	4087 (99.6)	1715 (99.8)	2372 (99.5)
Mental health diagnosis^g^			
Yes	3479 (84.8)	1571 (91.4)	1944 (81.5)
No	624 (15.2)	147 (8.6)	441 (18.5)
Evidence of OUD or OUD in remission^g^			
Yes	2436 (59.4)	1191 (69.3)	1245 (52.2)
No	1667 (40.6)	527 (30.7)	1140 (47.8)
Other substance use^g^			
Yes	2969 (72.4)	1428 (83.1)	1561 (65.5)
No	1134 (27.6)	290 (16.9)	824 (34.5)
MOUD during pregnancy (anytime during the 9 mo prior to delivery)			
Yes	2137 (52.1)	1074 (62.5)	1063 (44.6)
No	1966 (47.9)	644 (37.5)	1322 (55.4)
MOUD at delivery (within 30 d before delivery)			
Yes	1889 (46)	941 (54.8)	948 (39.7)
No	2214 (54)	777 (45.2)	1437 (60.3)
Known injection drug use^h^			
Yes	1848 (45)	955 (55.6)	893 (37.4)
No	97 (2.4)	39 (2.3)	61 (2.6)
Missing	2158 (52.6)	724 (42.1)	1431 (60.0)
Injection related infection (ever)			
Yes	1087 (26.5)	549 (32.0)	572 (24.0)
No	3016 (73.5)	1169 (68.0)	1813 (76.0)

Abbreviations: GED, general educational development; HBV, hepatitis B virus; HIV, human immunodeficiency virus; HMO, health maintenance organization; *ICD*, *International Classification of Diseases, Ninth Revision* or *International Statistical Classification of Diseases and Related Health Problems, Tenth Revision*; MOUD, medications for opioid use disorder; NOWS, neonatal opioid withdrawal syndrome; NR, not reported; OUD, opioid use disorder; WCC, well-child care.

^a^ Appropriately tested defined as HCV antibody testing at ≥18 months of age or HCV RNA testing at ≥2 months of age.

^b^ The other category varies by individual Public Health Datawarehouse dataset and includes any non-Hispanic race that is not White, Black, or Asian. Asian or Pacific Islander and American Indian or other, non-Hispanic groups combined for maternal characteristics because totals between 1 and 10 in individual groups stratified by appropriate testing.

^c^ Values 1 to 10 were suppressed for privacy reasons per Department of Public Health policy.

^d^ Complementary values were suppressed to prevent calculation of the suppressed values.

^e^ Responses combined into yes category include: Yes; Yes, living in emergency shelter; Suspected based on address or *ICD* diagnostic code; Reported unstable housing or *ICD* code for housing instability; Not homeless but had been in the past.

^f^ Calculated using the Kotelchuck Index.^24^ This index, also known as the Adequacy of Prenatal Care Utilization (APNCU) Index, uses prenatal care initiation timing and number of prenatal care visits to categorize people into 4 categories of prenatal care utilization by the percentages shown with a reference of expected number of visits for a low-risk pregnancy.

^g^ See eTables 1 and 2 in Supplement 1 for codes and databases utilized to identify and define; these were not limited to diagnoses during pregnancy and could have occurred any time during the study period.

^h^ At time of HCV diagnosis, as noted on HCV case report form.

Among birthing parents (mean [SD] age, 29.1 [4.9] years), 3463 were non-Hispanic White (84.4%) and 3185 (77.6%) were publicly insured. Few had evidence of HIV or HBV coinfection (16 [0.4%] and 47 [1.1%], respectively). Only 2304 deliveries (56.2%) had HCV recorded in birth certificate data; 3264 deliveries (79.6%) had a claim for the birthing parent’s HCV diagnosis during their delivery hospitalization. Histories of housing instability or homelessness (3186 [77.6%]), incarceration (1530 [37.3%]), mental health diagnoses (3479 [84.8%]), and substance use disorders (2969 [72.4%]) were prevalent.

### Care Cascade for Children Exposed to HCV

**Figure 2.**
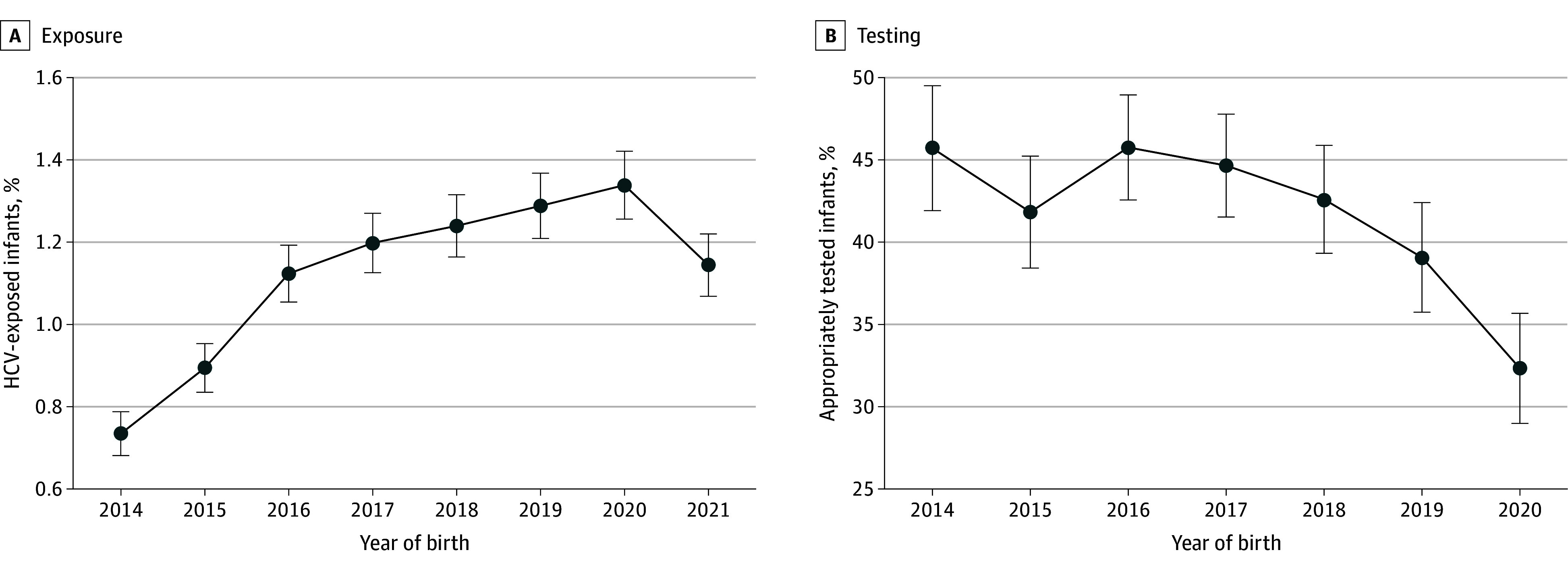
Line Chart of Hepatitis C Virus (HCV) Exposure Prevalence Among Live Births and Testing Among Exposed Infants by Year in Massachusetts A, The proportion of live births in Massachusetts included birthing parent with a probable or confirmed case of HCV, excluding those born to birthing parents treated for HCV prior to pregnancy; B, the percentage of children born between 2014-2020 to a birthing parent with a probable or confirmed case of HCV included infants who had appropriate HCV testing by December 2022 (defined as an HCV antibody test at age 18 months of life or older or an HCV RNA test at 2 months of life or older). Bars represent 95% CIs.

By December 2022, 1905 (46.4% of 4103 exposed children) had any HCV testing done (HCV RNA or antibody completed at any age), and 1718 (41.9%) completed appropriate testing for perinatal HCV exposure (RNA testing starting at age 2 months or antibody testing starting at age 18 months) (Figure 2A). Of those who completed appropriate testing, 837 (48.7%) had antibody testing alone, 532 (30.9%) had RNA testing alone, and 349 (20.3%) had both antibody and RNA testing. The proportion appropriately tested varied by county (range, 32.5%-48.5%) and by birthing hospital (27.1%-67.4%).

### HCV Testing Trends by Year and Age

Including all infants born 2014 through 2021 (not just those old enough for testing or treatment by 2022), 4548 infants were born to 3693 birthing parents with probable or confirmed HCV. The annual percentage of infants exposed to HCV (as proportion of all live births in Massachusetts) rose from 0.73% (509 births) in 2014 to 1.34% (700 births) in 2020 and decreased to 1.1% in 2021 (Figure 2A).

Appropriate HCV testing declined over time; 45.7% (95% CI, 41.9%-49.5%) of infants born in 2014 compared with only 32.3% (95% CI, 28.9%-35.7%) of infants born in 2020 were appropriately tested (Figure 2B). The proportion of infants receiving antibody testing alone increased over time, while RNA testing alone decreased (eFigure 1 in Supplement 1).

An infant’s first antibody test occurred during the recommended timeframe (between ages 18 and 23 months) for 35.5% of tested infants (eFigure 2A in Supplement 1). However, 37.7% were antibody tested before the recommended 18 months, and 26.8% were tested at age 2 years or older. Fifteen percent of RNA testing occurred before the recommended interval, 37.8% at 2 to 6 months of age, and very little after age 2 years (7.1%) (eFigure 2B in Supplement 1). Age at first HCV test (antibody or RNA) was highest for those born in 2019 (median [IQR] age, 18 [10-23] months) compared with those born in 2014 through 2017 (median [IQR] age: 2014, 12 [3-21] months; 2017, 16 [4-20] months) (eTable 3 in Supplement 1).

### Factors Associated With Appropriate HCV Testing

In univariable analysis, infants identified as non-Hispanic Asian or Pacific Islander (odds ratio [OR], 0.32; 95% CI, 0.17-0.61) or Hispanic (OR, 0.75; 95% CI, 0.61-0.91) had significantly lower odds of appropriate HCV testing compared with infants identified as White non-Hispanic (Table 2). Lower odds were also observed among infants residing in Berkshire County or with an older birthing parent, or among birthing parents who were born outside the US, identified as Black non-Hispanic, spoke a primary language other than English, had college or postgraduate education, or had private insurance.

**Table 2.** Factors Associated With Appropriate Testing of Infants Born Into a Birthing Parent With Probable or Confirmed Hepatitis C Virus (HCV)

Characteristics	Odds of appropriate testing, OR (95% CI)	
	Unadjusted^a^	Adjusted^b^
**Infant**		
Sex		
Male	1 [Reference]	NR
Female	1.03 (0.90-1.17)	NR
Race and ethnicity		
White, non-Hispanic	1 [Reference]	NR
American Indian or other, non-Hispanic^c^	0.66 (0.42-1.06)	NR
Asian or Pacific Islander, non-Hispanic	0.32 (0.17-0.61)	NR
Black, non-Hispanic	0.80 (0.61-1.04)	NR
Hispanic	0.75 (0.61-0.91)	NR
Residential county at birth		
Barnstable	0.94 (0.70-1.28)	0.75 (0.54-1.06)
Berkshire	0.63 (0.40-0.98)	0.45 (0.28-0.73)
Bristol	1.06 (0.83-1.35)	0.93 (0.71-1.23)
Dukes	NR^d^	NR^d^
Essex	0.78 (0.60-1.01)	0.83 (0.62-1.12)
Franklin	0.68 (0.38-1.21)	0.50 (0.27-0.95)
Hampden	0.80 (0.59-1.09)	0.70 (0.49-1.00)
Hampshire	1.09 (0.64-1.86)	0.90 (0.49-1.65)
Middlesex	1 [Reference]	1 [Reference]
Nantucket	NR^d^	NR^d^
Norfolk	1.25 (0.93-1.67)	1.43 (1.02-2.01)
Plymouth	1.03 (0.80-1.34)	1.19 (0.88-1.60)
Suffolk	1.16 (0.87-1.55)	1.27 (0.90-1.80)
Worcester	0.79 (0.62-1.02)	0.71 (0.54-0.95)
Preterm birth		
No	1 [Reference]	NR
Yes	1.09 (0.91-1.30)	NR
Diagnosis of NOWS on birth certificate		
No	1 [Reference]	NR
Yes	1.23 (1.02-1.48)	NR
Unknown	0.85 (0.68-1.06)	NR
Any WCC captured between 18-36 mo of age		
No	1 [Reference]	1 [Reference]
Yes	8.17 (6.26-10.66)	10.47 (7.82-14.00)
**Birthing parent**		
Age at delivery, mean (SD) per 1-y difference	0.97 (0.96-0.98)	0.99 (0.97-1.00)
Age group at delivery		
19-25 y	1 [Reference]	NR
≤18 y	NR	NR
26-35 y	0.91 (0.78-1.06)	NR
>35 y	0.60 (0.47-0.76)	NR
Unknown	NR	NR
Race and ethnicity		
White, non-Hispanic	1 [Reference]	1 [Reference]
Asian or Pacific Islander, American Indian, other, non-Hispanic^c^	0.55 (0.40-0.75)	0.85 (0.45-1.62)
Black, non-Hispanic	0.59 (0.80-0.59)	0.65 (0.45-0.94)
Hispanic	0.63 (0.81-0.63)	0.86 (0.63-1.17)
Born outside the US		
No	1 [Reference]	1 [Reference]
Yes	0.42 (0.31-0.56)	0.83 (0.57-1.21)
Unknown	NR	NR^d^
Insurance at delivery		
Public	1 [Reference]	1 [Reference]
Private	0.47 (0.40-0.56)	0.76 (0.62-0.93)
Unknown	1.08 (0.56-2.08)	3.32 (0.96-11.47)
Language spoken		
English	1 [Reference]	NR
Spanish or other	0.45 (0.29-0.71)	NR
Refused or unknown	1.07 (0.65-1.78)	NR
Highest level of education		
High school degree or GED	1 [Reference]	NR
No high school degree	0.92 (0.76-1.12)	NR
Associate or Bachelor’s degree	0.51 (0.40-0.65)	NR
Postgraduate education	0.13 (0.07-0.24)	NR
Other or unknown	0.94 (0.80-1.10)	NR
Ever experienced housing instability or homelessness^e^		
No	1 [Reference]	1 [Reference]
Yes	2.41 (2.01-2.89)	1.52 (1.22-1.90)
Unknown or missing	NR^d^	NR^f^
Ever Incarcerated		
No	1 [Reference]	NR
Yes	1.43 (1.25-1.64)	NR
**Clinical or birth data**		
Prenatal Care Adequacy (Kotelchuck Index)^g^		
Adequate (80%-109%)	1 [Reference]	NR
Inadequate (<50% of expected visits)	1.56 (1.31-1.86)	NR
Intermediate (50%-79%)	1.05 (0.83-1.33)	NR
Intensive (≥110%)	1.11 (0.93-1.32)	NR
Missing or unknown	1.60 (1.16-2.21)	NR
Prenatal care site		
Private physician’s office	1 [Reference]	1 [Reference]
Community health center	1.25 (0.97-1.60)	1.36 (1.0-1.84)
HMO	0.91 (0.59-1.39)	1.19 (0.73-1.96)
Hospital clinic	1.31 (1.11-1.54)	1.27 (1.03-1.56)
Other	1.47 (0.77-2.82)	1.69 (0.77-3.73)
Unknown	1.02 (0.74-1.41)	NR
Diagnosis of HBV		
No	1 [Reference]	NR
Yes	0.80 (0.43-1.49)	NR
Unknown	NR	NR
Diagnosis of HCV on birth certificate		
No	1 [Reference]	NR
Yes	2.31 (2.02-2.64)	NR
Unknown	NR	NR
Diagnosis of HCV in APCD within 30 d of delivery		
No	1 [Reference]	1 [Reference]
Yes	6.95 (5.54-8.72)	5.4 (4.19-6.96)
Diagnosis of HIV		
No	1 [Reference]	NR
Yes	NR^d^	NR
Mental health diagnosis		
No	1 [Reference]	NR
Yes	2.44 (1.98-3.00)	NR
Evidence of opioid use disorder		
No	1 [Reference]	NR
Yes	2.05 (1.78-2.35)	NR
Other substance use		
No	1 [Reference]	NR
Yes	2.61 (2.22-3.06)	NR
MOUD during pregnancy (anytime 9 mo prior to delivery)		
No	1 [Reference]	1 [Reference]
Yes	2.07 (1.81-2.37)	1.29 (1.09-1.51)
MOUD at delivery (during the month of or before delivery)		
No	1 [Reference]	NR
Yes	1.83 (1.60-2.09)	NR
Known injection drug use^h^		
No	1 [Reference]	NR
Yes	1.74 (1.13-2.68)	NR
Missing	1.31 (0.84-2.02)	NR
Injection related infection		
No	1 [Reference]	NR
Yes	1.49 (1.29-1.73)	NR

Abbreviations: APCD, Massachusetts All-Payer Claims Database; GED, general educational development; HBV, hepatitis B virus; HCV, hepatitis C virus; HMO, health maintenance organization; HIV, human immunodeficiency virus; *ICD*, *International Classification of Diseases, Ninth Revision* or *International Statistical Classification of Diseases and Related Health Problems, Tenth Revision*; MOUD, medications for opioid use disorder; NOWS, neonatal opioid withdrawal syndrome; NR, not reported; WCC, well-child care.

^a^ Appropriately tested defined as HCV antibody testing at ≥18 months of age or HCV RNA testing at ≥2 months of age.

^b^ Adjusted for all variables shown with clustered standard errors by birthing parent.

^c^ The other category varies by individual Public Health Datawarehouse dataset and includes any non-Hispanic race that is not White, Black, or Asian. Asian or Pacific Islander and American Indian or other non-Hispanic groups were combined for maternal characteristics because totals of between 1 and 10 in individual groups stratified by appropriate testing.

^d^ Values 1 to 10 were suppressed for privacy reasons per Department of Public Health policy.

^e^ Responses combined into yes category included: Yes; Yes, living in emergency shelter; Suspected based on address or *ICD* diagnostic code; Reported unstable housing or *ICD* code for housing instability; Not homeless but had been in the past.

^f^ Complementary values are suppressed to prevent calculation of the suppressed values; unknown or missing groups were excluded from regressions if the small numbers were unlikely to reveal meaningful results or prevented the model from converging.

^g^ Calculated using the Kotelchuck Index.^24^

^h^ At time of HCV diagnosis, as noted on HCV case report form.

Infants with NOWS, whose birthing parent’s HCV diagnosis was documented, or who had a well-child visit captured between ages 18 to 36 months were significantly more likely to be appropriately tested. Higher odds of appropriate pediatric testing were also associated with a birthing parent who had inadequate prenatal care, hospital clinic–based prenatal care, an MOUD prescription during pregnancy, or who were ever noted to have incarceration, housing instability, a mental health diagnosis, OUD (active or in remission), or another substance use disorder.

In multivariable analysis, having any well-care visits between ages 18 to 36 months (adjusted OR [aOR], 10.47; 95% CI, 7.82-14.00) or the birthing parent’s HCV diagnosis recorded within 30 days of delivery (aOR, 5.40; 95% CI, 4.19-6.96) had the highest increase odds in the association with appropriate testing (Table 2). MOUD during pregnancy was also associated with higher odds of appropriate testing (aOR, 1.29; 95% CI, 1.09-1.51). Infants born to birthing parents who identified as Black non-Hispanic (compared with White non-Hispanic: aOR, 0.65; 95% CI, 0.45-0.94) or had private insurance (compared with public insurance: aOR, 0.76; 95% CI, 0.62-0.93) had lower odds of appropriate testing. Residence in certain counties outside the Boston metropolitan area also were associated with significantly lower testing odds (Berkshire: OR, 0.45; 95% CI, 0.28-0.73; Worcester: OR, 0.71; 95% CI, 0.54-0.95).

### Sensitivity Analysis

Restricting to children with continued well-care captured through age 18 to 36 months, results were consistent with the primary analysis. However, multivariable associations were attenuated.

### Care Cascade for Children With HCV Infection

**Figure 3.**
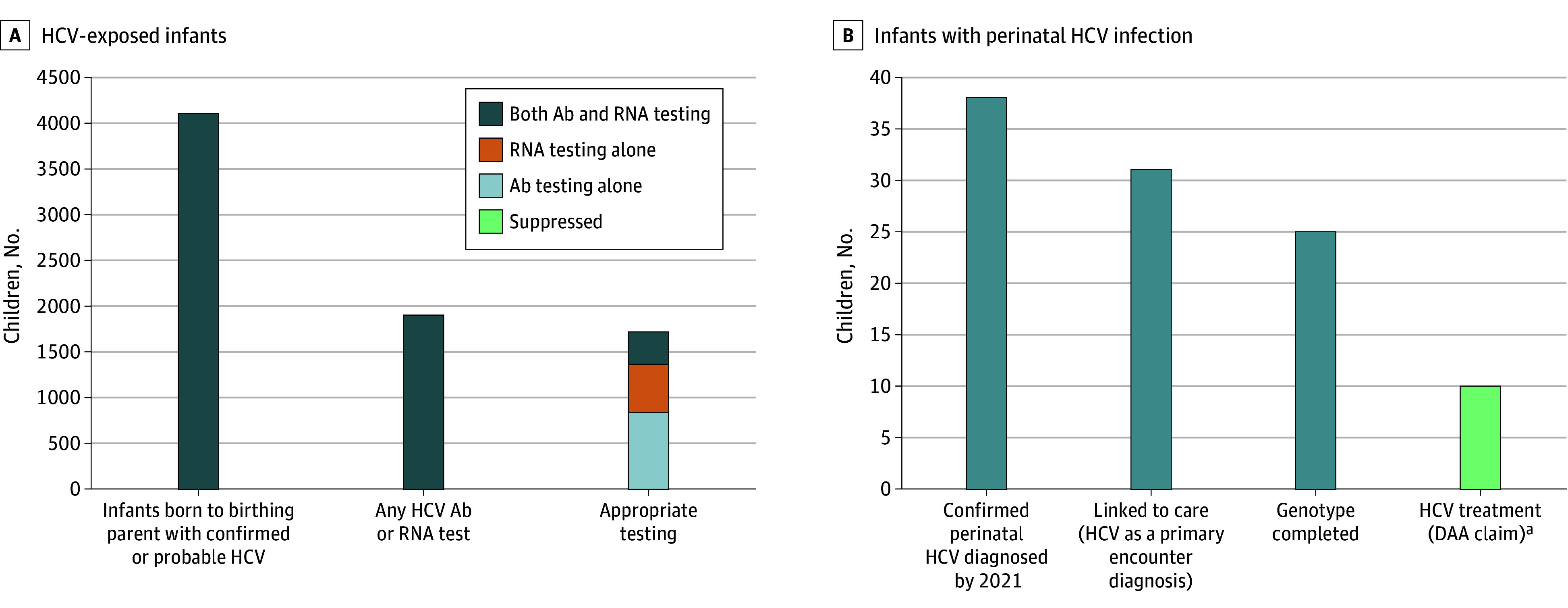
Bar Chart of Hepatitis C Virus (HCV) Care Cascade Among Infants Exposed to HCV and With Perinatal HCV Infection Ab indicates antibody. A, the number and proportion of children born to birthing parents with a probable or confirmed case of HCV who had any testing and appropriate testing (defined as an HCV antibody test at age 18 months of life or older or an HCV RNA test at 2 months of life or older); B, the care cascade for children with a confirmed perinatal HCV case reported by 2021 and who were born by June 2019 such that they were at least age 3.5 years by study end in 2022, and therefore could have been treated. ^a^Because the total treated was less than 11 children, values 1 through 10 were suppressed per applicable Department of Public Health privacy requirements; the suppressed bar indicates that the total could be any number between 1 and 10.

Of 1577 children born from 2014 through 2019 and appropriately tested by 2021, 40 (2.5%) had confirmed perinatal HCV by case report to the DPH. Of the 38 children with perinatal HCV who were aged 3.5 years or older by study end in 2022, 31 (81.6%) linked to care, 25 (65.8%) had genotype testing, and fewer than 11 (<29.0%) had evidence of HCV treatment (Figure 3B).

## Discussion

To our knowledge, this is the first statewide analysis of factors associated with appropriate HCV testing of children perinatally exposed to HCV to include robust covariates on linked birthing parent-infant dyads and linkage to care and treatment for children in the DAA era. Fewer than half of perinatally exposed children received appropriate testing, HCV testing decreased over time, and few children were treated (fewer than 11 of 38 eligible). One of the factors with the most increased odds of appropriate HCV testing was documentation of the birthing parent’s HCV within 30 days of delivery. Factors related to parental HCV risk (ie, substance use treatment and housing instability) were also associated with increased HCV testing odds in exposed infants. Conversely, characteristics that correlate with better health care access but lower HCV risk—private insurance and prenatal care at a private physician’s office—had significantly lower odds of appropriate HCV testing. Essentially, pediatricians are more likely to test infants when they know or suspect the birthing parent has HCV.

We observed geographic variation in HCV testing practice. Infants residing in several rural counties were significantly less likely to be tested. Structural barriers to testing may pose considerable obstacles, such as lacking local pediatric HCV specialists, on-site phlebotomy access, transportation, or linkage between electronic health records systems. Studies to understand barriers to testing, current testing rates, and factors associated with higher or lower testing odds, educational initiatives on the recent guidelines, electronic medical record alerts, and pediatric note templates that include HCV testing results alongside all other prenatal testing results could improve guideline familiarity, remind pediatricians to assess for HCV, and empower pediatricians to take charge of testing and addressing these barriers.^25,26,27^

MOUD and insurance associations were consistent with previous smaller studies.^10,12,16^ Race and ethnicity associations were more nuanced. While having a birthing parent who identified as American Indian or other non-Hispanic categories, Asian or Pacific Islander, non-Hispanic Black, or Hispanic was associated with decreased testing odds in univariable analyses, only Black race was associated with decreased testing odds in adjusted models. This is consistent with findings in a recent Tennessee statewide analysis,^16^ but contrary to results in other studies.^11,14,15,17^ This study adjusted for more sociodemographic and geographic factors than the other studies, suggesting race and ethnicity rather than being independent risk factors for testing may be instrumental variables associated with social vulnerability and inequities in care, including structural racism.

HCV testing rates decreased over time. While the COVID-19 pandemic likely affected testing access, proportions did not increase prior to the pandemic and did not show evidence of recovery in 2022. This study period preceded the 2023 CDC recommendations to test exposed children between 2 and 6 months of age. We observed early RNA testing adoption, similar to other studies,^10,15,18^ although most RNA testing occurred after 6 months of age. Earlier and more widespread RNA testing could improve testing rates by reducing loss to follow-up.

While 82% of treatment-eligible children were linked to care, few had evidence of treatment, consistent with 12% to 14% treated in recent pediatric HCV treatment studies.^15,28^ While these data reflect as few as 1 years of treatment eligibility (the first pangenotypic regimen was approved for 3-to-11-year-olds in 2021), treatment logistics in children are also challenging. No liquid formulations exist, only granule or pellet formulations, which taste bitter if chewed and require administration with food. A 2025 study^29^ found 51% of children referred for HCV treatment could not swallow pills upon referral. While 92% achieved SVR, approaching clinical trial SVR rates,^30^ not all settings have specialized pharmacy teams to facilitate medication approvals and swallowing practice. Additionally, there are relatively few pediatric HCV specialists. Programs such as the Kentucky Hepatitis Academic Mentorship Program could expand HCV treatment into pediatric primary care as occurs in adults,^31^ but this requires pediatrician buy-in and time to incorporate treatment into their practice. This study defines a baseline upon which such programs could build.

Despite 2020 CDC recommendations that all pregnant people be HCV tested each pregnancy, less than 50% of pregnant people are screened for HCV in that timeframe. Therefore, it is likely that many more infants are untested due to unknown maternal infection status.^4,32,33^ It is critical that (1) we test all pregnant people for HCV, (2) prenatal clinicians communicate HCV results to pediatricians, and (3) pediatricians check prenatal HCV testing results routinely for all children, regardless of background or perceived HCV risk, to ensure all exposed children are identified and tested.

### Strengths and Limitations

A study strength was our ability to link birthing parent-infant dyads across several statewide databases that form a population-level dataset. However, the single-state nature limits generalizability. Detailed covariate information can help determine applicability of findings to other settings and populations. We could not adjust for foster care status, although collinearity with other key variables may have precluded its inclusion in the multivariable model. This study is limited by inability to capture care for infants who moved out of state. However, 84% of included children had well-care visits captured between 18 to 36 months of age when HCV testing was recommended. Sensitivity analyses demonstrated similar findings when limiting to this group. We lacked laboratory results and therefore defined HCV infection by case reports, which may have misclassified individuals’ current infection status or missed results not reported to the state or diagnoses that occurred out of state or before 2011. Including infants born to those with probable HCV cases also overestimates exposed infants and therefore underestimates the proportion infected. However, excluding cases without confirmatory RNA testing would alternatively underestimate those exposed and overestimate the proportion infected. We were unable to analyze factors associated with linkage to care or treatment (given only 38 confirmed perinatal HCV infections with the suppression of values less than 10) or to determine which children spontaneously cleared infection; therefore, the percentage treated is likely an underestimate. However, even if 50% had spontaneously cleared, still less than 50% would have been treated.

## Conclusions

This cohort study of US infants perinatally exposed to HCV found that few children with known exposure are tested or treated and that documentation around delivery or suspicion of a birthing parent’s HCV infection is associated with higher testing odds. Eliminating perinatal HCV transmission is a US Viral Hepatitis National Strategic Plan priority. The CDC perinatal testing recommendations may improve early identification, but will only be successful if HCV exposure is clearly communicated and barriers to pediatric testing and treatment are addressed.

## Notes

Supplement 1. eMethods. eTable 1. MA Public Health Datawarehouse Datasets from which variables were abstracted eTable 2. Codes used in data extraction eFigure 1. Number of HCV-exposed infants appropriately tested for HCV, by year and test type eTable 3. Age at first HCV antibody or RNA test eFigure 2. Age at First HCV antibody or RNA test

Supplement 2. Data Sharing Statement
