# Supplementary material for: Hepatitis C Virus Testing in Perinatally Exposed Children
Source: JAMA Netw Open. 2026 Apr 17;9(4):e260743. doi: 10.1001/jamanetworkopen.2026.0743 (PMC13090848; doi:10.1001/jamanetworkopen.2026.0743)
Supplement: Supplement 1. — eMethods. eTable 1. MA Public Health Datawarehouse Datasets from which variables were abstracted eTable 2. Codes used in data extraction eFigure 1. Number of HCV-exposed infants appropriately tested for HCV, by year and test type eTable 3. Age at first HCV antibody or RNA test eFigure 2. Age at First HCV antibody or RNA test [file jamanetwopen-e260743-s001.pdf]

Supplemental Online Content

Epstein RL, Munroe S, Erdman EA, et al. Hepatitis C Virus Testing in Perinatally Exposed Children in Massachusetts, 2014-2021. *JAMA Netw Open.* 2026;9(3):e260743. doi:10.1001/jamanetworkopen.2026.0743

eMethods.

eTable 1. MA Public Health Datawarehouse Datasets from which variables were abstracted

eTable 2. Codes used in data extraction

eFigure 1. Number of HCV-exposed infants appropriately tested for HCV, by year and test type

eTable 3. Age at first HCV antibody or RNA test

eFigure 2. Age at first HCV antibody or RNA test

This supplemental material has been provided by the authors to give readers additional information about their work.

© 2026 Epstein RL et al. *JAMA Network Open*.

eMethods: Description of Massachusetts Department of Public Health (DPH) Public Health Data Warehouse (PHD)

This study combines a wide array of datasets sourced from the Massachusetts Public Health Data Warehouse (PHD) housed by the Massachusetts Department of Public Health (DPH). Analysis was conducted between January 2023 – February 2025 and final data reflect the PHD updates of August-September 2024.

The PHD is an integrated repository of public health and healthcare datasets, linking state and local municipality data to facilitate comprehensive population-level analyses.[1] This study utilizes a wide array of the PHD datasets to investigate Hepatitis C Virus (HCV) exposures, infections, and care trajectories among women of reproductive age with Opioid Use Disorder (OUD) or OUD in remission. Individual datasets and brief descriptions are listed below and individual variables abstracted from each dataset in eTables 1-2 below. For additional details please see: https://www.mass.gov/info-details/public-health-data-warehouse-phd-technical-documentation.

The study integrates medical claims, vital statistics data, HCV case reporting data, and substance use treatment data to determine HCV exposures, cases, and care, and covariates.

Primary Datasets utilized in this study:

1. **MAVEN:** Hepatitis C virus (HCV) and HIV surveillance data is maintained by the Massachusetts Virtual Epidemiologic Network (MAVEN), the DPH's integrated disease surveillance and case management system. Such information is reported to DPH pursuant to 105 CMR 300.00, and includes residency, demographics, clinical presentation, and risk factors from laboratories, healthcare providers, and service organizations, and, when possible, the individuals themselves.
2. **APCD:** The All-Payer Claims Database (APCD), maintained by the Center for Health Information and Analysis, is a comprehensive repository of medical and pharmacy claims from both commercial insurers and public programs such as Medicaid/MassHealth. This database integrates claims from medical carriers as well as specialty services, including mental health and substance use treatment. In addition to detailed records on healthcare expenditures and service utilization, the APCD contains demographic and enrollment information on individual plan members, which can be linked to provider and insurance product data, such as coverage and plan type. Alongside hospital and addiction treatment records, the APCD facilitates analysis of emergency medical services and pharmacy claims, offering a robust framework for assessing healthcare access and utilization across Massachusetts. Since 2016, self-funded private insurance plans have not had mandated reporting to the APCD, and many of these plans have not reported data to the APCD for 2017 on. For example, while they comprised 20% of individual member eligibility records in 2015, they represented only 5% of those records in 2019.
3. **Case Mix:** The Acute Care Hospital Case Mix dataset, maintained by the Center for Health Information and Analysis, contains comprehensive data on patient demographics, clinical presentation, and services provided for inpatient stays, emergency visits, and outpatient care for acute care hospitals.

© 2026 Epstein RL et al. *JAMA Network Open*.

4. **BSAS:** The Bureau of Substance Addiction Services (BSAS), part of DPH, oversees and licenses addiction treatment providers in Massachusetts and is the payer of last resort for uninsured and underinsured residents. BSAS collects substance use and treatment data from clients or providers, which can offer key insights into substance use disorder care. The DPH has created variables to identify if a client is receiving buprenorphine, naltrexone, or methadone, though there are clients for whom it is unclear which MOUD is prescribed based on the changing codes and services over time.
5. **MATRIS:** The Massachusetts Ambulance Trip Record Information System (MATRIS) captures data on EMS incidents from licensed ambulance providers across the state, and is managed by the DPH. Required under 105 CMR 170.000 (Emergency Medical Services System), these records follow the National Emergency Medical Services Information System (NEMSIS) standards and offer critical insights into emergency care and access for Massachusetts residents. Data include calls for which no hospital or emergency department visit occurred. The DPH has developed a variable to determine if a trip involves an opioid overdose based on an algorithm developed in conjunction with the CDC.
6. **Vital Statistics Data**: The Massachusetts Department of Public Health's Registry of Vital Records and Statistics (RVRS) maintains official records of all births, deaths, and reportable fetal deaths involving Massachusetts residents. Birth data are submitted by licensed birthing hospitals, birthing centers, and local clerks for home births, and include detailed information from both medical staff and parents. Death records include demographic details and the official cause and manner of death as determined by physicians or medical examiners. Fetal death records capture stillbirths at or beyond 20 weeks gestation or weighing at least 350 grams, aligning with national reporting standards set by the CDC's National Center for Health Statistics.

The DPH also created 3 datasets that integrate data from several separate datasets:

1. **Spine Demographics Dataset:** This dataset searches all PHD datasets to create the best possible demographic variables for each ID. Variables include information on race, ethnicity, sex, year of birth, housing status, disability status, preferred language, education level, and other additional details.
2. **Spine MOUD Dataset**: This dataset provides episode-level data on medications for OUD (MOUD). The MOUD dataset includes details on medication type and start and end dates for each unique treatment episode for each person. This dataset consolidates information contained in the PHD from the APCD, BSAS, Department of Corrections, and the Prescription Monitoring Program, specifically focusing on Buprenorphine, Methadone, and Naltrexone treatments. In this project, we censored the Spine MOUD dataset to only include records that occurred after a patient's OUD diagnosis date as some MOUD (specifically Naltrexone) can also be used to treat alcohol use disorder.
3. **Spine Overdose Dataset:** Finally, the Spine Overdose Dataset includes data on both fatal and non-fatal opioid overdoses, sourced from the Case Mix datasets, the APCD, MATRIS, and Vital Statistics death records. It captures overdose incidents by ZIP code, age, gender, and race, enabling detailed analysis of opioid-related health outcomes.

© 2026 Epstein RL et al. *JAMA Network Open*.

Description of exclusion of self-funded and no APCD claim:

For the outcome of total HCV-exposed infants in the state, all linked dyads with HCV exposure were included. However, for testing and care cascade/treatment outcomes, we applied exclusion criteria to ensure proper capture in the datasets. Children who transitioned to a self-funded insurance plan after March 2016 and subsequently ceased to have identifiable data in the state's All-Payer Claims Database (APCD) from that period onwards were excluded. Additionally, we required that children possess at least one claim (medical, dental, or pharmacy) documented in the APCD. These criteria were applied to ensure the cohort comprised individuals whose healthcare utilization remained consistently trackable within the APCD, thereby enhancing the accuracy and relevance of analyses conducted in this study.

© 2026 Epstein RL et al. *JAMA Network Open*.

© 2026 Epstein RL et al. *JAMA Network Open*.

eTable 1: MA Public Health Datawarehouse Datasets from which variables were abstracted

Variable	Infant or Birthing Parent	Data Source	Description
Sex	Infant	Spine Demographic Dataset	Male, Female, or Unknown
Preterm birth	Infant	Birth Records Infant/Child: Registry of Vital Records and Statistics	Obstetric estimate of gestation at delivery (completed weeks); Birth is considered preterm if delivery occurred prior to 37 weeks' gestation
Infant race/ethnicity	Infant	Spine Demographic Dataset	Race/Ethnicity categorized by the MA DPH using a tiered algorithm to search 25 PHD datasets to determine the most accurate race/ethnicity, prioritizing those with self-reported values
Residential county at birth	Infant	Birth Records Infant/Child: Registry of Vital Records and Statistics	County of residence at birth
Diagnosis of NOWS	Infant	Birth Records Infant/Child: Registry of Vital Records and Statistics	Neonatal abstinence syndrome indicated on birth certificate
Any WCC captured between 18-36 months of age	Infant	All Payer Claims Data – Medical	Any well-child visit ICD code between 18-36 months of age
Age at Delivery	Birthing Parent	Birth Records Birthing Parent: Registry of Vital Records and Statistics	Birthing Parent's age in years
Parent Race/Ethnicity	Birthing Parent	Spine Demographic Dataset	Race/Ethnicity categorized by the MA DPH
Born outside the U.S.	Birthing Parent	Spine Demographic Dataset	Was the person born outside the US?
Insurance at delivery	Birthing Parent	Birth Records Birthing Parent: Registry of Vital Records and Statistics	Type of insurance at delivery
Language Spoken	Birthing Parent	Birth Records Birthing Parent: Registry of Vital Records and Statistics	Language preferred for speaking about health
Highest level of education	Birthing Parent	Birth Records Birthing Parent: Registry of Vital Records and Statistics	Highest grade of elementary or secondary school that the birthing parent completed
Ever recorded to have housing instability or homelessness	Birthing Parent	Spine Demographic Dataset	Has the person been identified between 2011-2023 as homeless at least once at the time of contact?
Ever Incarcerated	Birthing Parent	Spine Demographic Dataset	Has person been incarcerated in MA's DOC or Berkshire, Essex, Franklin, Hampden, Hampshire, Middlesex, Norfolk, Plymouth, Suffolk, or Worcester HOC

© 2026 Epstein RL et al. *JAMA Network Open*.

Variable	Infant or Birthing Parent	Data Source	Description
Kotelchuck Index	Birthing Parent	Birth Records Birthing Parent: Registry of Vital Records and Statistics	Adequacy of Prenatal Care Utilization Index
Prenatal Care Site	Birthing Parent	Birth Records Birthing Parent: Registry of Vital Records and Statistics	Primary prenatal care site
Diagnosis of Hepatitis B Virus on birth certificate	Birthing Parent	Birth Records Birthing Parent: Registry of Vital Records and Statistics	Birthing Parent infections - Hepatitis B
Diagnosis of Hepatitis C Virus on birth certificate	Birthing Parent	Birth Records Birthing Parent: Registry of Vital Records and Statistics	Birthing Parent infections - Hepatitis C
Claim for Hepatitis C Virus during delivery hospitalization	Birthing Parent	All Payer Claims Data – Medical	ICD claim of Hepatitis C Virus Infection within 30 days of delivery date
HIV Diagnosis by Case Report	Birthing Parent	HIV Incidence (MAVEN)	Birthing Parent infections - Human Immunodeficiency Virus
Mental Health Diagnosis	Birthing Parent	All Payer Claims Data – Medical	ICD diagnosis of any mental health disorder
Evidence of Opioid Use Disorder (OUD) or OUD in Remission	Birthing Parent	All Payer Claims Data – Medical & Pharmacy (APCD); Case Mix; PMP; BSAS; MATRIS; Vital Statistics (birth and death tables); Spine Medications for Opioid Use Disorder (MOUD) Dataset; Spine Overdose Dataset	See eTable 2 for full code list; additionally if BSAS had notation of opioid use. Full description of each component of OUD and MOUD definition is described elsewhere.[2,3]
Other substance use	Birthing Parent	All Payer Claims Data – Medical; Bureau of Substance Addiction Services	See eTable 2 for full code list; additionally included if BSAS had notation of non-opioid substance use
MOUD during pregnancy	Birthing Parent	Spine MOUD Dataset	Any instance of buprenorphine, methadone, or naltrexone prescription anytime in the 9 months prior to delivery.
MOUD at delivery	Birthing Parent	Spine MOUD Dataset	Any instance of buprenorphine, methadone, and naltrexone use during the delivery month or the month prior.
Known injection drug use	Birthing Parent	Hepatitis C (MAVEN)	Has the case ever injected drugs not prescribed by a doctor?
Injection-related infection	Birthing Parent	All Payer Claims Data – Medical	See eTable 2 for full code list
HCV Case Reports	Both	Hepatitis C (MAVEN)	See main text for details of whether confirmed or confirmed and probable cases used in each situation.

© 2026 Epstein RL et al. *JAMA Network Open*.

Abbreviations: MA DPH, Massachusetts Department of Public Health; NOWS, Neonatal Opioid Withdrawal Syndrome; WCC, Well-child care; MAVEN, Massachusetts Virtual Epidemiologic Network; OUD, Opioid Use Disorder; Case Mix, (the Acute Care Hospital Case Mix); PMP, Prescription Monitoring Program; BSAS, Bureau of Substance Addiction Services; MATRIS, Massachusetts Ambulance Trip Record Information System; MOUD, Medications for Opioid Use Disorder; DOC, Department of Corrections.

© 2026 Epstein RL et al. *JAMA Network Open*.

eTable 2: Codes used in data extraction

Variable	ICD-9/10, HCPCS, and CPT Codes
Opioid Use Disorder (OUD) or OUD in remission; includes codes for opioid use or dependence, overdose, and medications for OUD; capture of any of these codes met our definition of OUD or OUD in remission	30400, 30401, 30402, 30403, 30470, 30471, 30472, 30473, 30550, 30551, 30552, 30553, F1110, F1111, F11120, F11121, F11122, F11129, F1113, F1114, F11150, F11151, F11159, F11181, F11182, F11188, F1119, F1120, F1121, F11220, F11221, F11222, F11229, F1123, F1124, F11250, F11251, F11259, F11281, F11282, F11288, F1129, F1193, F1199, 9701, 96500, 96501, 96502, 96509, E8500, E8501, E8502, T400X1A, T400X2A, T400X3A, T400X4A, T400X1D, T400X2D, T400X3D, T400X4D, T401X1A, T401X2A, T401X3A, T401X4A, T401X1D, T401X2D, T401X3D, T401X4D, T402X1A, T402X2A, T402X3A, T402X4A, T402X1D, T402X2D, T402X3D, T402X4D, T403X1A, T403X2A, T403X3A, T403X4A, T403X1D, T403X2D, T403X3D, T403X4D, T404X1A, T404X2A, T404X3A, T404X4A, T404X1D, T404X2D, T404X3D, T404X4D, T40601A, T40601D, T40602A, T40602D, T40603A, T40603D, T40604A, T40604D, T40691A, T40692A, T40693A, T40694A, T40691D, T40692D, T40693D, T40694D, T40411A, T40411D, T40412A, T40412D, T40413A, T40413D, T40414A, T40414D, T40421A, T40421D, T40422A, T40422D, T40423A, T40423D, T40424A, T40424D, G2067, G2068, G2069, G2070, G2071, G2072, G2073, G2074, G2075, G2076, G2077, G2078, G2079, G2080, H0020, HZ81ZZZ, HZ84ZZZ, HZ91ZZZ, HZ94ZZZ, J0570, J0571, J0572, J0573, J0574, J0575, J0592, J2315, Q9991, Q9992, S0109
Other Substance Use	2910, 2911, 2912, 2913, 2914, 2915, 2918, 29181, 29182, 29189, 2919, 30300, 30301, 30302, 30390, 30391, 30392, 30500, 30501, 30502, 76071, 9800, 3575, 4255, 53530, 53531, 5710, 5711, 5712, 5713, F101, F1010, F1012, F10120, F10121, F10129, F1013, F10130, F10131, F10132, F10139, F1014, F1015, F10150, F10151, F10159, F1018, F10180, F10181, F10182, F10188, F1019, F102, F1020, F1022, F10220, F10221, F10229, F1023, F10230, F10231, F10232, F10239, F1024, F1025, F10250, F10251, F10259, F1026, F1027, F1028, F10280, F10281, F10282, F10288, F1029, F109, F1090, F1092, F10920, F10921, F10929, F1093, F10930, F10931, F10932, F10939, F1094, F1095, F10950, F10951, F10959, F1096, F1097, F1098, F10980, F10981, F10982, F10988, F1099, T405X4A, 30421, 30422, 3056, 30561, 30562, 3044, 30441, 30442, 9697, 96972, 96973, 96979, E8542, F14, F141, F1410, F1412, F14120, F14121, F14122, F14129, F1413, F1414, F1415, F14150, F14151, F14159, F1418, F14180, F14181, F14182, F14188, F1419, F142, F1420, F1421, F1422, F14220, F14221, F14222, F14229, F1423, F1424, F1425, F14250, F14251, F14259, F1428, F14280, F14281, F14282, F14288, F1429, F149, F1490, F1491, F1492, F14920, F14921, F14922, F14929, F1493, F1494, F1495, F14950, F14951, F14959, F1498, F14980, F14981, F14982, F14988, F1499, F15, F151, F1510, F1512, F15120, F15121, F15122, F15129, F1513, F1514, F1515, F15150, F15151, F15159, F1518, F15180, F15181, F15182, F15188, F1519, F152, F1520, F1522, F15220, F15221, F15222, F15229, F1523, F1524, F1525, F15250, F15251, F15259, F1528, F15280, F15281, F15282, F15288, F1529, F159, F1590, F1592, F15920, F15921, F15922, F15929, F1593, F1594, F1595, F15950, F15951, F15959, F1598, F15980, F15981, F15982, F15988, F1599, T405, T436, T405XIA, T43601A, T43602A, T43604A, T43611A, T43621A, T43624A, T43631A, T43634A, T43641A, T43644A, 96970, 96972, 96973, 96979, 97081, 97089, E8542, E8543, E8552, T43691A, T43694A
HCV Diagnosis	7051, 7054, 707, 7041, 7044, 7071, B1710, B182, B1920, B1711, B1921
Injection-Related Infection	3642, 9884, 11281, 11504, 11514, 11594, 421, 4211, 4219, A382, B376, I011, I059, I079, I080, I083, I089, I330, I339, I358, I378, I38, T826, I39, 681, 6811,

© 2026 Epstein RL et al. *JAMA Network Open*.

	6819, 682, 6821, 6822, 6823, 6824, 6825, 6826, 6827, 6828, 6829, L030, L031, L032, L033, L038, L039, M000, M001, M002, M008, M009, 711, 7114, 7115, 7116, 7118, 7119, I800, I801, I802, I803, I808, I809, 451, 4512, 4518, 4519
Well-child visit	99381, 99391, 99381, 99391, 99381, 99391, 99382, 99392
**HCV Treatment**	**NDC Codes**
Direct-Acting Antivirals	00003021301, 00003021501, 61958220101, 61958180101, 61958180301, 61958180401, 61958180501, 61958150101, 61958150401, 61958150501, 72626260101, 00074262501, 00074262528, 00074262556, 00074262580, 00074262584, 00074260028, 72626270101, 00074308228, 00074006301, 00074006328, 00074309301, 00074309328, 61958240101, 61958220101, 61958220301, 61958220401, 61958220501, 00006307402, 51167010001, 51167010003, 59676022507, 59676022528, 00085031402
**HCV Tests**	**Common Procedural Terminology (CPT)**
HCV Antibody	G0472, 86803, 86804, 80074
HCV RNA	87520, 87521, 87522
HCV Genotype	87902, 3266F

Abbreviations: HCV, hepatitis C virus; NAS, Neonatal Abstinence Syndrome; WCC, Well-child care; MOUD, Medications for Opioid Use Disorder; ICD-9/10, International Classification of Diseases-9/10; HCPCS, Healthcare Common Procedure Coding System; NDC, National Drug Codes; CPT, Current Procedural Terminology Codes; OUD, opioid use disorder

© 2026 Epstein RL et al. *JAMA Network Open*.

eFigure 1: Number of HCV-exposed infants appropriately tested for HCV, by year and test type

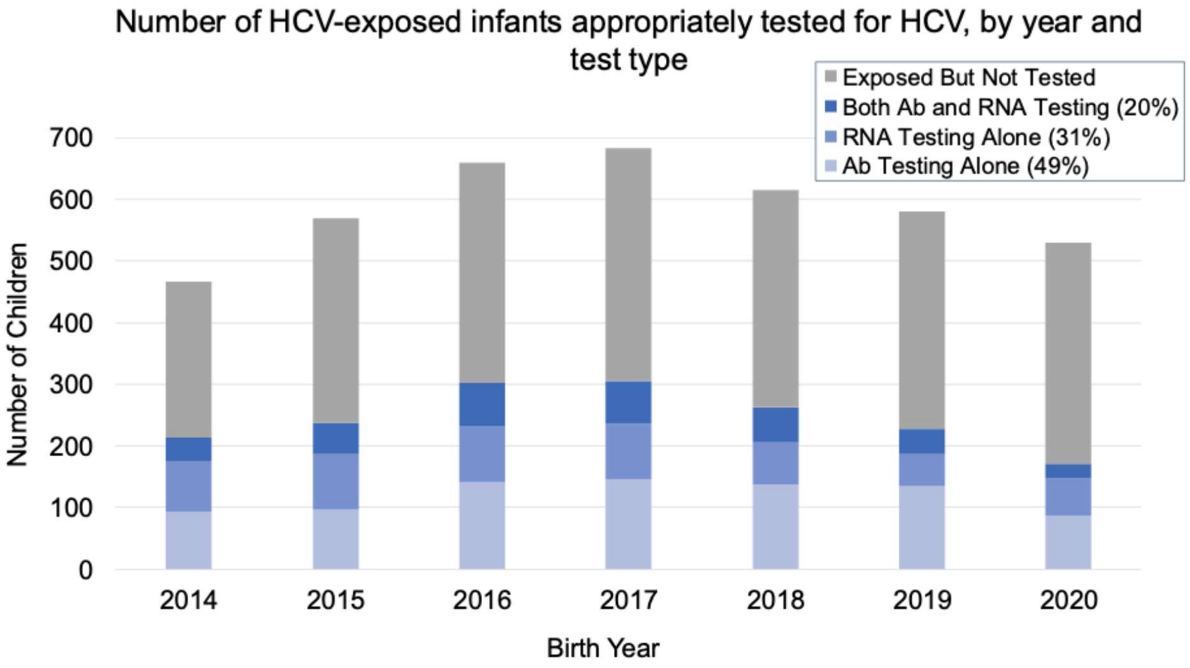

© 2026 Epstein RL et al. *JAMA Network Open*.

eTable 3: Age at first HCV antibody or RNA test

Year of Birth	Median (IQR) Age at First Test (months)	Mean Age at First Test (months)	p-value*
2014	12 (3-21)	15.5	0.004
2015	14 (4.5-20.5)	15.2	0.003
2016	16 (4-21.5)	15.9	0.05
2017	16 (4-20)	14.7	0.002
2018	18 (6-23.5)	15.7	0.36
2019	18 (10-23)	17.2	-
2020	18 (7-19)	14.6	0.11

*Kruskal-Wallis test comparing median month at first test by birth cohort year was significant overall and p-values shown are for the given birth year cohort compared to 2019 birth cohort, which had the highest mean age at first HCV antibody or RNA test

© 2026 Epstein RL et al. *JAMA Network Open*.

eFigure 2a: Age at First HCV Antibody Test

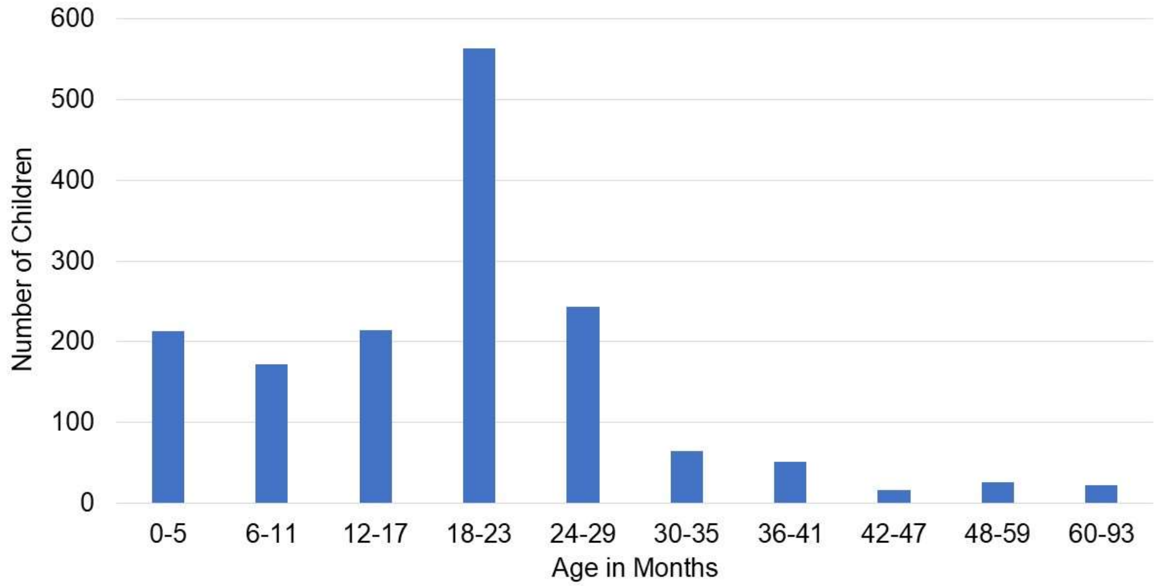

eFigure 2b: Age at First HCV RNA Test

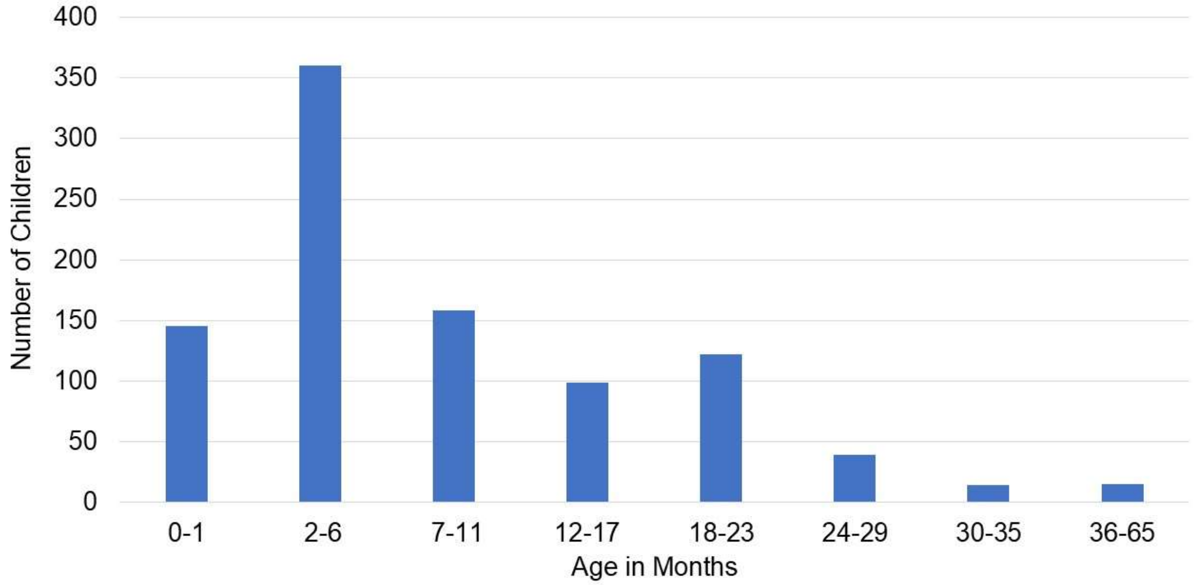

© 2026 Epstein RL et al. *JAMA Network Open*.

References:

1. Bharel M, Bernson D, Averbach A. Using Data to Guide Action in Response to the Public Health Crisis of Opioid Overdoses. *NEJM Catalyst*. 2020;1(5). doi:10.1056/CAT.19.1118

2. Bovell-Ammon BJ, Yan S, Dunn D, et al. Prison Buprenorphine Implementation and Postrelease Opioid Use Disorder Outcomes. *JAMA Netw Open*. 2024;7(3):e242732. doi:10.1001/jamanetworkopen.2024.2732

3. Public Health Data Warehouse (PHD) Technical Documentation | Mass.gov. Accessed January 19, 2025. https://www.mass.gov/info-details/public-health-data-warehouse-phd-technical-documentation

© 2026 Epstein RL et al. *JAMA Network Open*.
