# Supplementary material for: Hepatitis C Virus Testing in Perinatally Exposed Children
Source: JAMA Netw Open. 2026 Apr 17;9(4):e260743. doi: 10.1001/jamanetworkopen.2026.0743 (PMC13090848; doi:10.1001/jamanetworkopen.2026.0743)
Supplement: Supplement 2. — Data Sharing Statement [file jamanetwopen-e260743-s002.pdf]

Data Sharing Statement

Epstein. Hepatitis C Virus Testing in Perinatally Exposed Children in Massachusetts, 2014-2021. *JAMA Netw Open*. Published March 31, 2026. doi:10.1001/jamanetworkopen.2026.0743

Data
Data available: No

Additional Information
Explanation for why data not available: The data used in this study cannot be shared by the authors because the Massachusetts Public Health Data Warehouse (PHD) is restricted and managed by the Massachusetts Department of Public Health (DPH). Requests for access to this data source should be directed to the Massachusetts DPH (DPH.PHD@mass.gov). General information about the PHD, including technical documentation, is available at: https://www.mass.gov/public-health-data-warehouse-phd.
